# Evaluation of serum extracellular vesicles as noninvasive diagnostic markers of glioma

**DOI:** 10.7150/thno.33114

**Published:** 2019-07-09

**Authors:** Huayi Wang, Dengzhi Jiang, Wenzhe Li, Xiang Xiang, Jun Zhao, Bin Yu, Chen Wang, Zhaohui He, Ling Zhu, Yanlian Yang

**Affiliations:** 1 CAS Key Laboratory of Standardization and Measurement for Nanotechnology, CAS Key Laboratory of Biological Effects of Nanomaterials and Nanosafety, CAS Center for Excellence in Nanoscience, National Center for Nanoscience and Technology, Beijing 100190, China; 2 Department of Neurosurgery, The First Affiliated Hospital of Chongqing Medical University, 1 Friendship Road, Chongqing 400016, China; 3 Department of Chemistry, Tsinghua University, Beijing, 100084, China; 4 Department of Radiology, The First Affiliated Hospital of Chongqing Medical University, 1 Friendship Road, Chongqing 400016, China; 5 University of Chinese Academy of Sciences, 19 A Yuquan Rd, Shijingshan District, Beijing 100049, China; 6 Academy for Advanced Interdisciplinary Studies, Peking University, Beijing 100871, China

**Keywords:** extracellular vesicle, glioma, liquid biopsy, EGFR, *NLGN3*

## Abstract

**Rationale**: Glioma is the most common malignant primary brain tumor in the central nervous system (CNS). The lack of reliable noninvasive diagnostic and prognostic methods is one of the main reasons for the high mortality of glioma. Serum has become a useful biomarker for the diagnosis and prognosis prediction of glioma because extracellular vesicles (EVs) carry molecular components from their parental cells.

**Methods**: To detect EVs and perform molecular analysis of serum EVs, we established and optimized a microbead-assisted method based on flow cytometry and estimated the efficacy of EGFR protein expression and *NLGN3* and *PTTG1* mRNA in serum EVs from glioma patients (n=23) and healthy individuals (n=12). We evaluated the ability of EGFR^+^ EVs to differentiate high-grade and low-grade glioma patients and checked the correlation between EGFR in EVs and the ki-67 labeling index (LI) in the tumor tissue.

**Results**: We demonstrated that EGFR^+^ EVs are effective diagnostic and prognostic markers of glioma. The expression of EGFR in serum EVs can accurately differentiate high-grade and low-grade glioma patients, and EGFR in EVs positively correlates with ki-67 LI in the tumor tissue. We also showed the potential of *NLGN3* and *PTTG1* mRNA in EVs for detecting glioma patients.

**Conclusions**: We demonstrate that the protein expression of EGFR in serum EVs is an effective diagnostic marker of glioma. EGFR in EVs highly correlates with the malignancy of glioma. We also show the potential of NLGN3 and PTTG1 in EVs for detecting glioma. The optimized flow cytometry with the aid of microbead-based EV enrichment show its potential as a noninvasive method for the detection of glioma and will be beneficial to the management of glioma.

## Introduction

Glioma is the most common and malignant brain tumor 1. The five-year relative survival rate of a primary malignant brain and other CNS tumors is 35.0% in the US 2. The high morbidity and mortality of glioma is mainly due to its resistance to therapy and rapid tumor growth and invasion to the brain 1, 3, 4. The current diagnosis of glioma mainly relies on neuroimaging techniques, such as magnetic resonance imaging (MRI), which are expensive and time- and labor-consuming 5. Blood-based detection methods are convenient for the diagnosis of glioma and the dynamic monitoring of tumor progress during treatment and will help in diagnosis, prognosis evaluation, and studies on the mechanism of the progression, recurrence, and treatment resistance of glioma 6. In the past several years, liquid biopsy has gained increasing attention in glioma. For instance, changes (e.g., mutation and methylation) in the serum DNAs (ctDNAs), such as MGMT 7-9, EGFR 10, and PTEN 9, have been proven to be effective biomarkers of glioma and may have the potential for the diagnosis of glioma. Circulating tumor cells (CTCs) have also been identified in glioma patients as markers for diagnosis and for evaluating treatment response 11-13. These advances demonstrated the importance of liquid biopsy as a noninvasive blood-based diagnostic method for glioma. However, due to the existence of blood brain barrier, the frequency of glioma CTCs released into blood has not been determined to date, limiting the widespread application of CTCs as routine diagnostic markers for glioma. The instability of ctDNA also demands effective storage methods and precise analytical methods.

Extracellular vesicles (EVs) are lipid bilayer-enclosed extracellular structures. EVs are formed by outward budding of the cell membrane or by an intracellular endocytic trafficking pathway 14-16. As a result of this biogenesis, EVs carry molecular information, such as the nucleic acids, membrane and cytosol proteins, and lipids, from source cells 17, facilitating the use of EVs as biomarkers for the diagnosis of diseases. Moreover, EVs are secreted by almost all cells, and exist extensively in body fluids, such as the blood, cerebrospinal fluid (CSF), and urine. Unlike CTCs that are rare in the blood, EVs are abundant in the blood. The amount of EVs is approximately 5
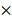
10^9^ vesicles per mL plasma 18. The lipid bilayer structure of EVs also protects the inside nucleic acids from degradation. These advantages make EVs desirable candidates as liquid biopsies for the diagnosis of tumors. Many studies have shown the application of EVs for the diagnosis of cancer 19, 20. Some of the studies focus on glioma 21, 22. RNA expression profiles in serum EVs were found to be different between the glioblastoma patients and controls 23. EGFR^+^ EVs were demonstrated to be able to differentiate glioma patients from healthy individuals and were shown to be indicators of treatment efficacy 24, 25. MGMT mRNA in EVs was shown to be a predictive marker for the treatment efficacy of glioma 24. MicroRNA (miRNA) was also found in CSF EVs from glioblastoma patients and was demonstrated to be a candidate biomarker for glioma 26. miRNA-21 in CSF was reported to be associated with the tumor grade of glioma 22. These studies suggested the diagnostic and prognostic value of EVs for glioma.

Numerous techniques have been developed to separate and enrich EVs. These methods include the use of immunomagnetic beads 19, 20, micro- or nanopillar arrays 27, nanopores 28, and acoustic nanofilter systems 29. The detection and molecular information analysis of EVs using flow cytometry was challenging because the nanoscale size of EVs exceeded the detection limit of traditional flow cytometry. To solve this problem, EVs were adhered onto microsized immunobeads and stained with antibodies targeting membrane protein markers on serum EVs to yield distinguishable signals of EVs by flow cytometry 30-32. Taking advantage of this technique, Melo et al. demonstrated the capability of glypican-1 (GPC-1)^+^ EVs in the early diagnosis of pancreatic cancer 33. Li et al. further proved the diagnostic value of HER2^+^ and EpCAM^+^ EVs for breast cancer 34. These studies demonstrated the validity of microbead-assisted flow cytometry in the detection and analysis of serum EVs.

We present a latex-bead assisted flow cytometry method for the detection and molecular analysis of EVs and examine the clinical value of this method in diagnosing and predicting malignancy in glioma patients. We focus on the expression of epidermal growth factor receptor (EGFR), which is a transmembrane tyrosine kinase that regulates cell proliferation, migration and differentiation, in EVs from glioma patients, as EGFR is a common marker for glioma. Increased gene amplification and protein overexpression of EGFR has been found in almost 50% of glioma patients 35, 36. The EGF-EGFR pathway was considered a critical pathway in the regulation of neovascularization of glioma tumor growth 37, 38. Some evidence also showed that the expression of EGFR has some relationship with glioma patient prognosis, although this conclusion has been contradicted by other studies, and the prognostic value of EGFR for glioma has not been demonstrated to date 39-41. We found high expression levels of EGFR in EVs derived from glioma cells and demonstrated that EGFR^+^ serum EVs can be used to diagnose glioma patients with high sensitivity and specificity. Moreover, we found that EGFR in serum EVs can accurately differentiate high-grade and low-grade glioma patients, and EGFR in EVs positively correlates with ki-67 labeling index (LI), which represents the percentage of ki-67-positive cells in the tumor tissue assessed by immunohistochemistry that has been shown to be associated with the high malignancy and poor outcome of tumors 42-44, indicating that EGFR in serum EVs can reflect the malignancy of glioma. We also demonstrate the efficacy of *NLGN3* and *PTTG1* mRNA in serum EVs to diagnose glioma patients. These results demonstrate the clinical significance of serum EVs in the diagnosis and prognosis prediction of glioma and will be beneficial for glioma cancer management.

## Materials and Methods

### Cell lines

The following human cell lines were used: U87MG (human glioblastoma cell), U251 (human glioma cell), and HA (human astrocytes). U87MG and U251 were supplied by Shanghai Institutes for Biological Sciences, Cell Bank of Chinese Academy of Sciences (http://www.cellbank.org.cn/), a branch of the Chinese Center for Type Culture Collection, and HA were supplied by Chinese Academy of Medical Sciences and Peking Union Medical College. U87MG cells were cultured in Minimum Essential Medium (MEM, Gibco) containing 10% fetal bovine serum (FBS, Gibco), 1% (v/v) streptomycin-penicillin, 1.5 g/L NaHCO_3_, and 0.11 g/L sodium pyruvate. DMEM (Gibco) condition medium containing 10% FBS, 1% (v/v) streptomycin-penicillin, and 1.5 g/L NaHCO_3_ was used for U251 culturing. HA were cultured in astrocyte basal medium (ScienCell) containing 2% (v/v) fetal bovine serum and 1% (v/v) astrocyte growth supplement. The cells were cultured in culture dishes (Corning, 430167) in a humidified atmosphere with 5% CO_2_ at 37 °C in a cell culture incubator. The condition medium was replaced every 2 days until the cells reached 80-90% confluence. To harvest the cells, trypsinization (0.25 w/v% trypsin) was used for U87MG cells and U251 cells, and trypsinization (0.05 w/v% trypsin) was used for HA cells.

### Clinical samples

The study was approved by First Affiliated Hospital of Chongqing Medical University (2017-032). Twenty-three glioma patients and 12 healthy donors who had no sign of serious disease and no surgery within the past 12 years were recruited on the basis of an institutional board approved protocol. All patients signed the informed consent form. Demographic details of the participants are summarized in Tables S1 and S2 in the supporting information. The pre-operative blood and tissue samples were collected on the day of operation, and the post-operative blood was collected one week after operation. Blood was collected in blood collection tubes and centrifuged at 2,500 g for 10 min to separate serum. Tissue and serum were then stored at -80 °C.

### EV isolation and extraction

Differential centrifugation was used for EV extraction as previously reported 33 with a few changes. Fifteen dishes of cells were cultured for EV isolation for each cell line. FBS-EV-free, EV-production medium was used when the cells reached a confluence of 75% - 80%, and the supernatant was collected after 24 h; there were approximately 300,0000 cells in each dish at this time point. Approximately 80 mL of medium was collected from 15 dishes, and the medium was centrifuged at 800 g for 5 min to discard large dead cells and then at 2,000 g for 10 min to eliminate large cell debris. The supernatant was filtered using a 0.2-μm pore filter (Millipore, USA) to discard large vesicles. The final supernatant was ultracentrifuged at 100,000 g for 2 h to pellet the small vesicles. (Beckman Optima XPN-100; rotor, 70 Ti; tube type, Beckman-355618; k-factor, 44; no brake.) The pellet was washed at 100,000 g for 2 h again in 26 mL PBS to eliminate contaminating proteins. The supernatant was abandoned, and the EVs were resuspended in PBS and stored at 4 °C or -80 °C. All the centrifugations were performed at 4 °C.

For EV isolation from patient serum, 250 μL serum was thawed on ice and diluted in 15 mL PBS. Then, the fluid was centrifuged at 2,000 g for 15 min. A 0.2-μm pore was used to filter the vesicles larger than 200 nm. After that step, serum were ultracentrifuged at 150,000 g overnight (at least 8h) at 4 °C. The pellet was washed in 26 mL PBS at 4 °C for 2 h. Finally, the EVs were resuspended in PBS.

### EV Protein Quantification

EV protein quantification was performed immediately after the EVs were extracted. Twenty microliters of EV suspension was lysed by adding 20 μL lysis buffer (Pierce™ IP Lysis Buffer, 87787) containing 1/100 (v/v) Protease Inhibitor (Halt™ Protease Inhibitor Cocktail, 87785) and Phosphatase Inhibitor (Pierce™ Phosphatase Inhibitor Mini Tablets, A32957). After vortexing for 1 min, the tubes were placed on ice and lysed for 0.5 h. Before protein quantification, 10 s sonication in ice water was performed twice with a 50 s break between each cycle. Protein quantification was performed using a BCA Protein Assay Kit (Solarbio, PC0020). The results were read by a microtiter plate (Molecular Devices, SpectraMax i3). After the protein concentration was calculated, the EV suspension was further diluted with PBS to a final concentration of 0.2 μg/μL and aliquoted and stored at -80 °C for subsequent flow cytometry, RT-PCR and TEM analysis. EV suspensions for Western blot analyses were mixed with loading buffer (NuPAGE™ LDS Sample Buffer, 4X, NP0007) and heated for denaturing electrophoresis at 70 °C for 10 min and then stored at -20 °C.

### Transmission electron microscopy

Ten microliters of suspension was placed on a 200-mesh grids (Beijing Zhongjingkeyi Technology Co., Ltd., China) and allowed to stand for 2 min. Ten microliters of 1% (w/w) uranyl acetate solution was used for negative staining and standing for 30 s. A Hitachi electron microscope (HT7700, Hitachi High-Tech, Japan) was used for characterization.

### Nanoparticle tracking analysis

NanoSight LM14 (NanoSight Technology, Malvern, UK) was used for concentration and size measurements. A syringe was used for sample injection, and the measurement was carried by a camera, while the camera setting was 16, the detection threshold setting was 7 and the acquisition time was 60 s. Between different samples, the chamber was washed 3 times using PBS. The data were collected by nanoparticle tracking analysis software (NTA version 2.3; Malvern Instruments, Malvern, UK).

### Saturation assay

One microliter of aldehyde latex beads was added to different amounts of EVs in PBS (total volume 100 μL), rotated for 30 min at 37 °C (220 rpm/min on Thermoshaker MS-100) and then rotated overnight at 4 °C. Blocking was performed with 100 mM glycine and 10% BSA. (1 h, 220 rpm/min on Thermoshaker MS-100) Washing was performed 3 times using 2% BSA for 15 min one time. Beads-EVs were stained with 200 μL 0.5 μg/mL FM^TM^4-64FX at 37 °C for 15 min (Thermo Fisher F34653, Lot No. 1915811) (220 rpm/min on Thermoshaker MS-100). Washing was performed 3 times using PBS for 15 min at a time (220 rpm/min on Thermoshaker MS-100). BD Accuri C6 was used for the samples test.

### Flow cytometry analysis

EVs in PBS suspension that were extracted from the cell lines or the serum were mixed with aldehyde latex beads (Thermo, 4 μm, A37304, with a concentration of 1.3*10^9^ beads/mL) at a ratio of 4 μg EVs /1 μL beads (1.3*10^6^ beads) and incubated for 10 min at room temperature with steady rotation (rotating speed 220 rpm/min on Thermo-Shaker MS-100). The consistency of the aldehyde latex beads for flow cytometry analysis was confirmed (Figure S1 in the supporting information). Then, 100 μL PBS was added to the tube and incubated at 4 °C overnight (more than 7 h). Then, 100 mM glycine and 10% BSA were added to stop the reaction. Then, 100 μL 2% BSA was used to wash the beads and centrifuged for 1 min at 14,800 g. Optimization of the formula of the blocking buffer is shown in Figure S2 in the supporting information. Next, 100 μL of primary antibody (anti-EGFR, Abcam, ab231, dilution 1/100 or anti-CXCR4, Abcam, ab1670, dilution 1/200) was added to the beads and incubated for 1 h at room temperature with continuous rotation (rotating speed 220 rpm/min). Antibodies were washed twice as mentioned above. Then, 100 μL of secondary antibody (anti-rat, ab150157, Abcam, dilution 1/1000 or anti-goat, ab150127, Abcam, dilution 1/1000) was added and incubated for 30 min with steady rotation (rotating speed 220 rpm/min) at room temperature. Then, the cells were washed twice as mentioned above. The control was a secondary control only. Flow cytometry was performed on BD Accuri C6.

### Western blots analysis

Bicinchoninic acid assay (BCA assay) relative protein quantification was used for normalization of sample loading. Proteins with 6 μg (EGFR, CD81, FGF-2, Flottin-1, Grp94, HSA), 8 μg (securin) or 10 μg (NLGN3) were loaded on per channel. Samples were separated using the assay of vertical slab polyacrylamide gel electrophoresis. After that, proteins were transferred onto polyvinylidene fluoride membranes (PVDF Invitrogen) using wet electrophoretic transfer. The membranes were blocked with 5% (w/v) skim milk and then incubated with primary antibody in 3 mL primary antibody dilution buffer. Next, the membranes were washed three times for 5 min each with 5 mL TBST followed by incubation with secondary IgG and HRP-linked antibody.

Western blot antibodies are listed below: Anti-EGFR, Abcam, ab52894, 1/5000. Anti-CD81, CST, 10037S, 1/1000. Anti-FGF2, Abcam, ab16828, 2 μg/ mL. Anti-Securin, Abcam, ab3350, 1/1000. Anti-HSA, Biorbyt, orb24991, 1/1000. Anti-Grp94, Sino Biological, 106461, 1/1000. Anti-Flotillin 1, Abcam, ab133497, 1/10000. Anti-CD81, CST, sc166029, 1/500. Anti-mouse IgG, HRP-linked Antibody, CST, #7076, 1/1000. Anti-rabbit IgG, HRP-linked Antibody, #7074, 1/1000.

### Real-time PCR

TRIzol was used for mRNA extraction and followed by the QuantScript RT Kit (QuantScript RT Kit Cat#KR103-04, Tiangen, China) to convert mRNA to cDNA. Quantitative reverse transcription PCR was performed on SuperReal PreMix Plus (SuperReal PreMix Plus-SYBR Green, Cat#FP205-02, Tiangen). Forward primer sequence for *PTTG1* mRNAs: F- 5'-GCTCTGTTCCTGCCTCAGAT-3', reverse primer for all *PTTG1* mRNAs R-5'-GAGAGGCACTCCACTCAAGG-3'. Forward primer sequence for *NLGN3* mRNAs: F- 5'-GGGAGTCCCCTTTCTGAAGC-3', reverse primer for all *NLGN3* mRNAs: R-5'-CCTTCATGGCCACACTGACT-3'. Forward primer sequence for *GAPDH* mRNAs: F-5'- GAGAAGGCTGGGGCTCATTT-3', reverse primer for all *GADPH* mRNAs: R-5'-AGTGATGGCATGGACTGTGG-3'.

## Results

### High expression of EGFR in the EVs derived from glioma cells

**Figure 1:**
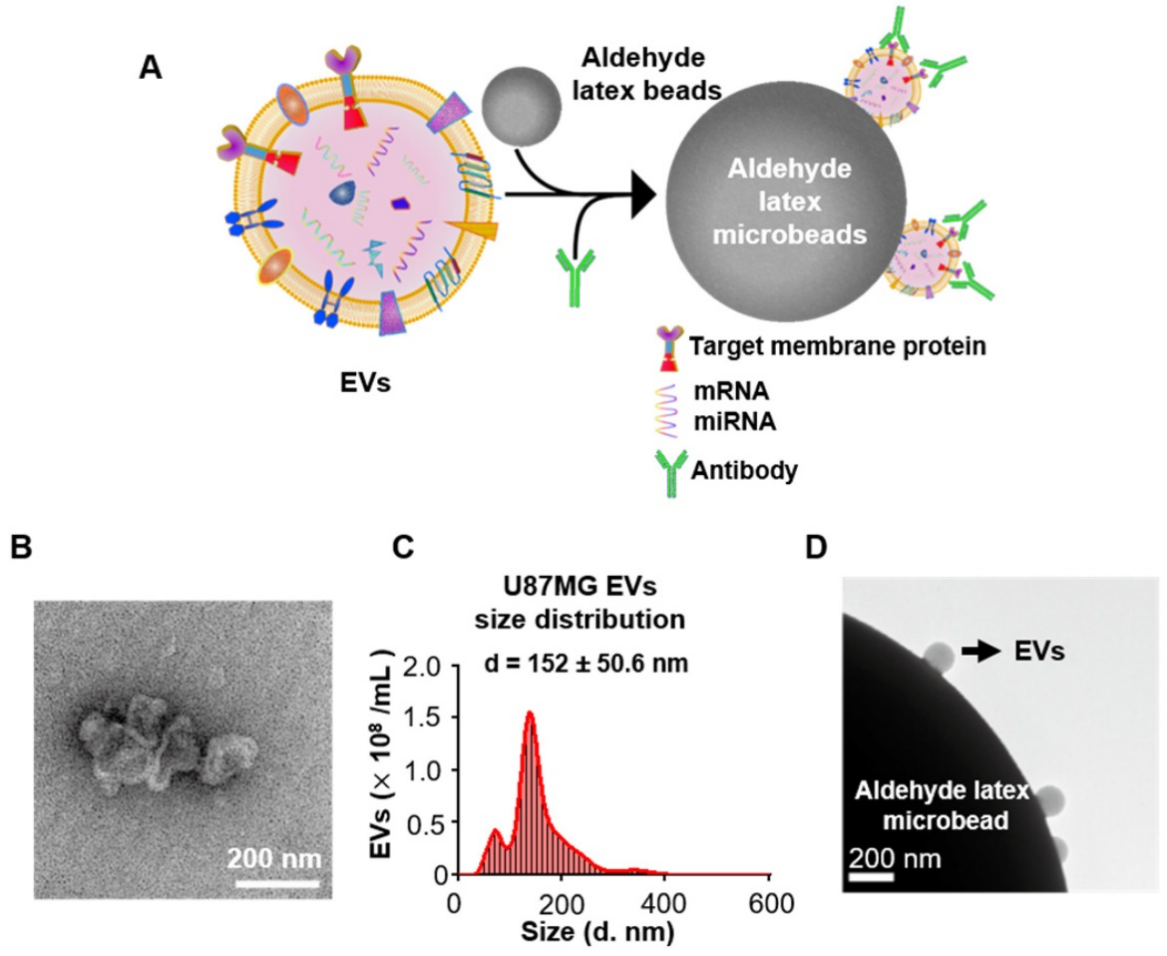
** EVs were extracted and enriched by microbeads.** (A) Schematic illustration of the enrichment and staining of EVs for flow cytometry analysis. EVs were attached to 4-μm aldehyde latex microbeads by aldimine condensation. Fluorescently labeled antibodies targeting the marker membrane proteins on the EVs were bound to the captured EVs for the following flow cytometry analysis. (B) Transmission electron microscopy (TEM) image of the EVs extracted from the U87MG cell line. (C) Size distribution of the EVs extracted from the U87MG cell line as analyzed by nanoparticle tracking analysis (NTA). (D) TEM verification of the EVs adhering to microbeads.

Flow cytometry has been commonly used to analyze the expression of membrane proteins on cells. However, the analysis of the expression of proteins in EVs through flow cytometry is scarce, since the nanoscale size of EVs is below the resolution of the traditional flow cytometer. To solve this problem, we attached the EVs on the surface of the aldehyde latex microbeads and labeled the captured EVs with fluorescent antibodies targeting the marker membrane proteins on the EVs. Thus, the microbeads covered with EVs are tested as a unit and are therefore detectable in the flow cytometry (Figure 1A).

**Figure 2:**
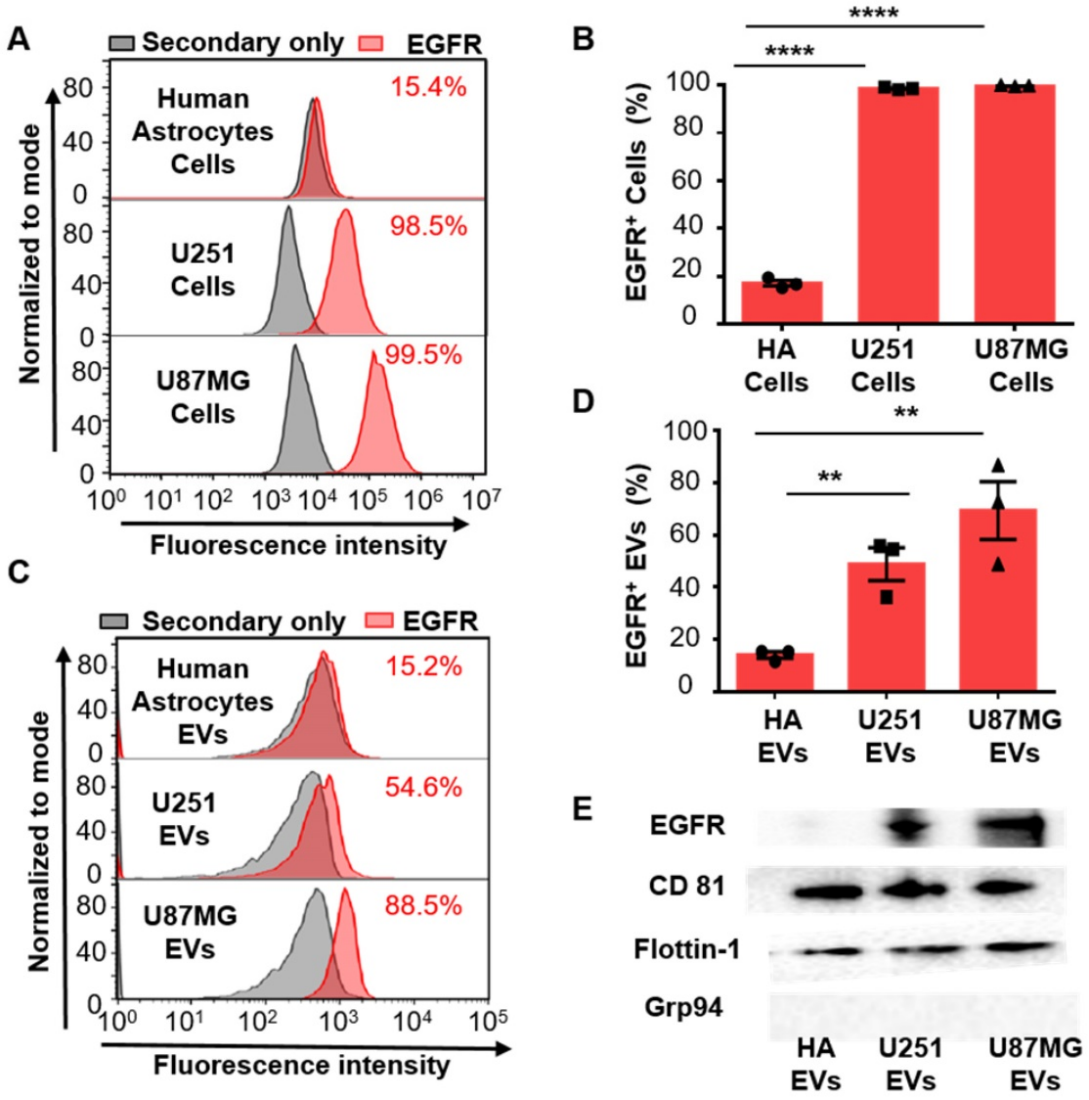
** Increased expression of EGFR in glioma cells and glioma cell-derived EVs.** (A) Representative flow cytometry histogram of the expression of EGFR in different cell lines. (Upper: Human Astrocytes (HA), Middle: U251, Lower: U87MG). (B) Percentage of EGFR expression in different cell lines analyzed by flow cytometry. Data are presented as the mean ± SEM. ****P <0.0001 (Student's t-test). (C) Representative flow cytometry histogram of EGFR^+^ EV beads in different cell lines. (Upper: HA, Middle: U251, Lower: U87MG). (D) Percentage of EGFR^+^ EVs in different cell lines analyzed by flow cytometry. Data are presented as the mean ± SEM. ** *P* < 0.01 (Student's t-test). (E) Western blot analysis of the expression of EGFR in EVs derived from different HA, U251 and U87MG cell lines. (Positive control: CD81 and Flotillin-1, negative control: Grp94, original blots are presented in Figure S5)

Since the overexpression of EGFR has been widely found in glioma 35, 36, we investigated if EGFR in EVs could also be taken as a biomarker for glioma. Two glioma cell lines (human glioblastoma cell line U87MG and human glioma cell line U251) and one normal cell line human astrocyte (HA) were chosen for comparison. Ultracentrifugation was used for EVs extraction from cell culture medium 33. Transmission electron microscopy (TEM) revealed a saucer-like morphology of the extracted vesicles (Figure 1B), which is characteristic for EVs 17, 45. Nanoparticle tracking analysis (NTA) showed that the vesicles had a size of 152 ± 50.6 nm (Figure 1C) with a concentration of 1.18 × 10^10^ /mL, corresponding to the reported size of EVs 46, 47. These results proved that the EVs were successfully extracted from the cell culture medium. The extracted EVs were then adhered onto the aldehyde latex microbeads and stained with anti-EGFR and the fluorescent secondary antibody for the subsequent flow cytometry analysis. The enrichment of EVs by the aldehyde latex microbeads was confirmed by TEM, which showed the adsorption of EVs on the microbeads (Figure 1D). To ensure that the enrichment of EVs on the microbeads was saturated, we performed a saturation assay to evaluate the saturation concentration of the microbeads. Different quantities of EVs were bound on the beads, and captured EVs were labeled with FM^TM^4-64FX, a dye to label EV membranes. The fluorescence was quantified by flow cytometry. The enrichment of EVs from different cell lines (U87MG and U251) on the beads was tested. The resulting saturation curve showed that the saturation ratio turned out to be ~1 μg EVs/1 μL beads (1.3*10^6^ beads) (Figure S3, Table S3). This finding was also supported by the TEM characterization of EVs bound on the beads at different EV/bead ratios that showed significantly increased amounts of EVs bound on the beads at 2~3 µg EVs/1 µL beads compared to 0.3 µg EVs/1 µL beads, and a similar density of EVs bound on the beads between 2 μg EVs/1 μL beads and 3 μg EVs/1 μL beads (Figure S4), suggesting that the EV/bead ratio of above 1 μg EVs/1 μL beads was sufficient for the saturation of the beads. Therefore, we used the EV/bead ratio of 4 μg EVs/1 μL beads (1.3*10^6^ beads) in the flow cytometry analysis to obtain the saturated enrichment of EVs on the microbeads. The expression of EGFR in the cell lines and the cell-derived EVs was analyzed by flow cytometry. As expected, the expression of EGFR was significantly higher in the glioma cell lines U87MG (unpaired Student's t-test, ***P < 0.0001) and U251 (unpaired Student's t-test, ***P < 0.0001) than in the normal HA cell line (Figure 2A, 2B), which was in line with the previous studies showing that EGFR was highly expressed in glioma cells 48, 49. Similarly, the expression of EGFR in the cell-derived EVs was significantly higher in the glioma cells U87MG (unpaired Student's t-test, **P < 0.01) and U251 (unpaired Student's t-test, **P < 0.01) than in the normal HA cells (Figure 2C, 2D), in the same tendency as the expression of EGFR in the parental cell lines, indicating that the expression of EGFR in the EVs was associated with the expression in the parental cells. These differences were further verified by Western blot analyses showing a significant band of EGFR in EVs extracted from U87MG and U251 and a negligible band in HA cells (Figure 2E). We also observed significant bands of the EV-characteristic proteins CD81 and Flotillin-1 and negligible expression of the cell-derived protein Grp94 in the EVs, further confirming the purity of the extracted EVs. (Figure 2E, Figure S5A, B). Western blotting showed the expression of Grp94 in the cell lysates, while no expression of this protein was observed in the EVs extracted from the cells, confirming the validity of Grp94 as a negative control (Figure S5B).These results, together with the flow cytometry analysis, demonstrated that EGFR in EVs could accurately show the expression of EGFR in the parental cells, and the expression level of EGFR was significantly higher in the glioma cells than in the normal cells, suggesting that EGFR in EVs could be taken as a candidate diagnostic marker for glioma.

### EGFR^+^ EVs as a diagnostic marker for glioma

**Figure 3:**
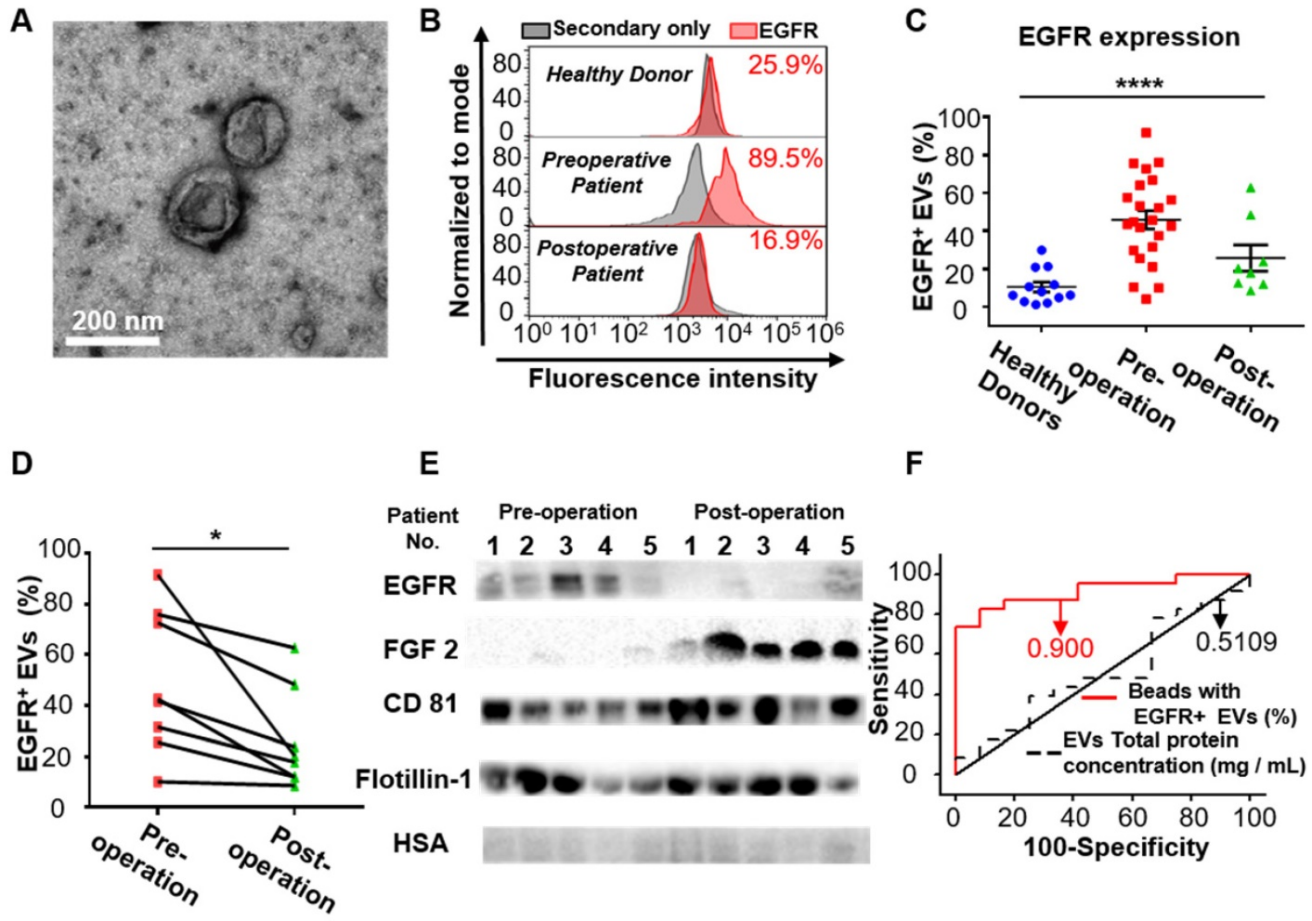
** EGFR^+^ EVs are noninvasive biomarkers for glioma**. (A) TEM image of the EVs extracted from the patients' serum. (B) Representative flow cytometry histogram of EGFR EV beads. Negative control: secondary antibody only (upper: healthy donor, middle: preoperative glioma patient, lower: postoperative glioma patient). (C) Percentages of EGFR^+^ EVs in healthy donors (n = 12), preoperative (n = 23) and postoperative patients (n = 8) glioma patients. Data are presented as the mean ± SEM. ****P < 0.0001 (one-way ANOVA). (D) Paired comparison of the percentage of EGFR^+^ EVs in individual glioma patients pre- and post-operation (paired comparison of the expression of EGFR in serum EVs from glioma patients pre- and post-operation. *P < 0.05 (paired t-test). (E) Western blot analysis of the expression of EGFR and FGF2 in EVs in glioma patients pre- and post-operation. (Positive control: CD81 and Flotillin-1, negative control: HSA, original blots are presented in Figure S5). (F) ROC curve showing the discriminative ability of EGFR^+^ EVs in differentiating glioma patients (n = 23) from healthy individuals (n = 12). The area under the curve (AUC) is annotated, and the detailed information is in Table S2 in the supporting information.

After demonstrating the high expression of EGFR in the EVs derived from glioma cells, we examined whether EGFR in serum EVs could be used to diagnose glioma. Twenty-three glioma patients and 12 healthy donors were enrolled for comparison. EVs were extracted from the serum of glioma patients and the control individuals by differential centrifugation 33. The size distribution and morphology of EVs extracted from human sera was characterized by TEM and NTA (Figure 3A, Figure S6 in the supporting information), demonstrating the successful extraction of EVs from human sera. Flow cytometry results indicated that the expression level of EGFR in EVs was significantly higher in the glioma patients compared to the healthy donors and was dramatically decreased one week after surgery (****P < 0.0001, one-way ANOVA, Figure 3C). To verify the validity of the flow cytometry-based EV analysis and to exclude the influence of different antibodies on the measurements, we compared two different EGFR antibodies and found that they exhibited similar measurements and positive correlations (Figure S7, Table S4). We further performed paired comparisons of the expression of EGFR in EVs pre- and post-operation in each of the 8 glioma patients who underwent surgical resection. A significant decrease in the expression of EGFR in EVs was found post-operation in each patient (paired Student's t-test, *P < 0.05, Figure 3D, Table S5), and the average decrease ratio after surgery was 0.440 ± 0.213 (Table S5). The remaining expression of EGFR might be due to wound healing after surgery, which has been shown to be related to the upregulation of growth factors. These results were further confirmed by Western blot analysis showing a significant reduction in the intensity of the EGFR band after surgery (Figure 3E). High expression of CD81 and Flotillin and negligible expression of human serum albumin (HSA) suggested the purity of the EVs from patient sera (Figure 3E).Western blotting showed the expression of HSA in human sera while no expression of this protein in the EVs isolated from sera, confirming the validity of HSA as a negative control (Figure S5). Interestingly, we found significantly increased expression of fibroblast growth factor 2 (FGF 2) in the serum EVs in glioma patients after surgery (Figure 3E). We attributed it to wound healing after surgery, as FGF has been reported to be upregulated after injury 50, 51. We further performed receiver operating characteristic (ROC) analysis to evaluate the discriminatory efficacy of EGFR^+^ EVs in distinguishing glioma patients and control individuals. We found that EGFR^+^ EVs exhibited high sensitivity (86.96%) and specificity (83.7%) in identifying glioma patients with an area under the curve (AUC) of 0.900 (95% CI of 0.8064 ~ 0.9936, *P* <0.0001, Figure 3F, Table S6 in the supporting information), indicating the high diagnostic value of EGFR^+^ EVs in detecting glioma. It has been reported that the total concentration of proteins in EVs could be used as an indicator for breast cancer 52 and prostate cancer 33. Therefore, we also tested the efficacy of the total concentration of proteins in EVs in diagnosing glioma. However, the diagnostic value of total protein concentration in EVs was inferior to the expression level of EGFR in EVs (AUC 0.5109, 95% CI 0.3089 ~ 0.7128,* P* = 0.9170, Figure 3F, Table S6 in the supporting information).

### EGFR in serum EVs correlates with the malignancy of glioma

**Figure 4:**
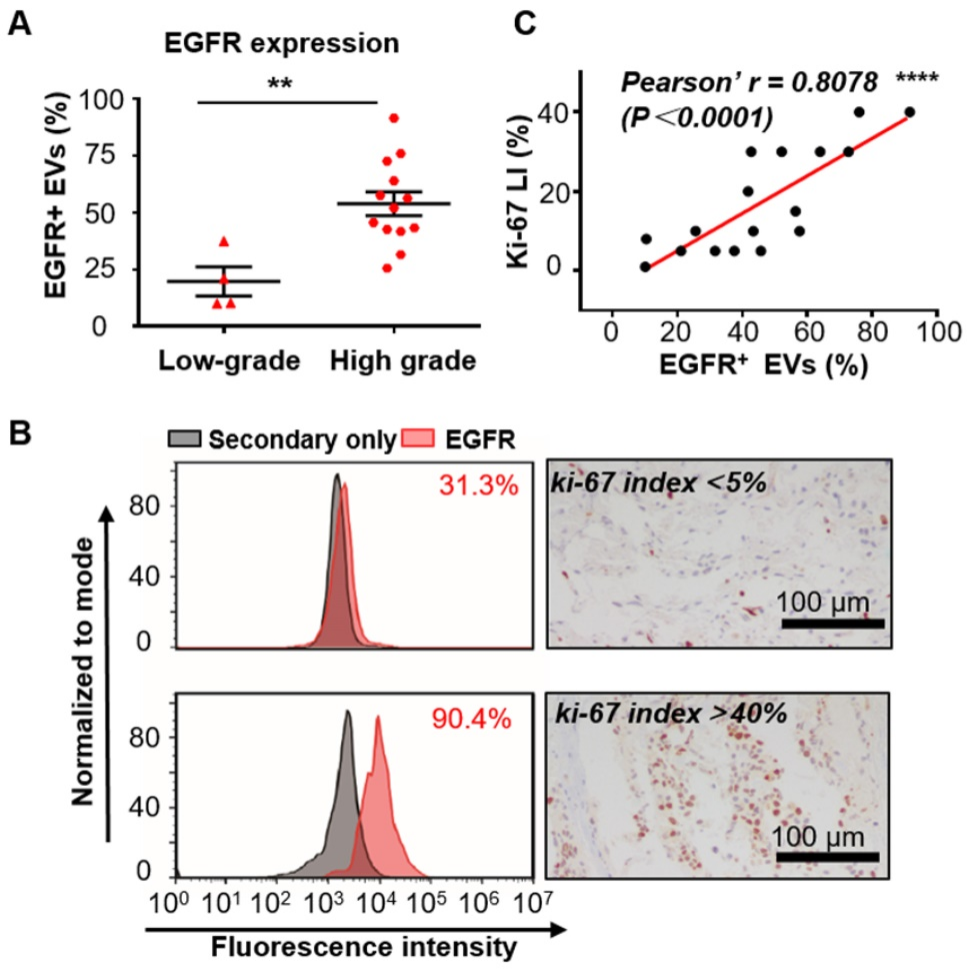
** Expression of EGFR in serum EVs correlates with the malignancy of glioma.** (A) Percentage of EGFR^+^ EVs in low-grade (n = 4) and high-grade (n =13) glioma patients (**P < 0.01, Student's t tests). (B) Comparison of the expression of EGFR in EVs tested by flow cytometry and ki67-LI tested by IHC in two glioma patients. (C) Pearson correlation analysis of (*r* = 0.8078, ****P < 0.0001).

Since increased expression of EGFR in tumor tissues has been reported to be associated with the malignancy and poor prognosis of glioma 37, we tend to test if EGFR in serum EVs could also reflect the malignancy of glioma. The expression level of EGFR was analyzed in the serum EVs from glioma patients with different grades diagnosed on the basis of the World Health Organization (WHO) classification 53. Seventeen glioma patients who underwent surgical resection (4 low-grade (grade II) and 13 high-grade (grade III and IV)) were enrolled for comparison. Flow cytometry analysis demonstrated that the expression level of EGFR in EVs was significantly higher in the high-grade glioma patients than in the low-grade ones (**P < 0.01, unpaired Student's t-test, Figure 4A), suggesting a correlation between EGFR in serum EVs and tumor malignancy. These results corresponded to previous studies showing that the expression of EGFR in tumor tissues increased with WHO grade 54. As ki-67 LI in tumor tissues is a widely accepted proliferation marker and a hallmark of tumor in the clinic 42, we also investigated the association between EGFR in EVs and ki-67 LI. Ki-67 is a nuclear protein that shows high expression in the active phases of the cell cycle but is absent in the resting cells. This protein represents the proliferating activities of the cells. Ki-67 LI, the percentage of ki-67-positive cells examined normally by immunohistochemical (IHC) staining, has been shown to be associated with the high malignancy and poor outcome of tumors 42-44. A positive correlation between ki-67 LI and the expression level of EGFR in tumor tissues has been reported in gastric cancer patients 55 and non-small-cell lung cancer (NSCLC) patients 56, 57. Moreover, a high value of ki-67 LI has also been reported to be correlated with EGFR mutations in NSCL patients and to be a prognostic indicator for NSCLC 56. These studies suggested a high correlation between ki-67 and EGFR. However, studies investigating the relationship between ki-67 and EGFR in glioma patients are scarce. Fraser et al. reported a moderate correlation between the expression of EGFR and ki-67 LI in the tumor tissues in canine gliomas (*r* = 0.47, *P* = 0.007) 58. We compared ki-67 LI between high-grade and low-grade patients. As expected, ki-67 Li was significantly higher in high-grade patients than in low-grade patients (Figure S8). We analyzed the relationship between EGFR in EVs and ki-67-LI in 17 patients with reported ki-67 LI and found a strong correlation between them (representing flow cytometry histogram and IHC staining of ki67 are shown in Figure 4B). Person correlation analysis showed a Pearson's r of 0.8078 (****P < 0.0001, Figure 4C), suggesting a strong correlation between EGFR in EVs and ki-67 LI. These results showed a strong relationship between EGFR in EVs and the proliferation of glioma, further demonstrating the capability of EGFR^+^ EVs as an indicator for tumor malignancy in serum noninvasive diagnoses.

It is well-known that the chemokine stromal cell derived factor-1 (CXCL12) C-X-C chemokine receptor 4 (CXCR4) pathway plays important roles in cell migration and is responsible for the progression and invasion of tumors 59, 60. The overexpression of CXCR4 has been found to be an indicator of tumor progression in various types of tumors, including glioma 60, 61. Therefore, we also measured the expression level of CXCR4 in serum EVs from glioma patients at different stages. Flow cytometry analysis showed that the expression level of CXCR4 in EVs was higher in high-grade (III & IV) glioma patients than in low-grade (II) ones (Figure S9 in the supporting information), although the difference was not statistically significant.

### *PTTG1* mRNA and *NLGN3* mRNA in EVs as diagnostic markers for glioma

**Figure 5:**
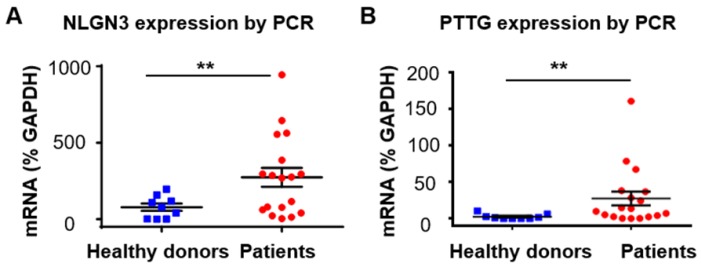
*NLGN3* and* PTTG1* mRNA in serum EVs as diagnostic markers for glioma. (A) *NLGN3* mRNA in EVs from glioma patients (n =18) and healthy donors (n =9) (**P < 0.01, Student's t tests). (B) *PTTG1* mRNA in EVs from glioma patients (n =18) and healthy donors (n =9) (**P < 0.01, nonparametric, Mann-Whitney test).

After exploring the diagnostic value of proteins in serum EVs for glioma, we also investigated the value of mRNA in serum EVs in diagnosing glioma. We focused on two mRNA markers, neuroligin-3 (*NLGN3*) and pituitary tumor transforming gene1 (*PTTG1*). Neuroligins are a family of synaptic cell-adhesion proteins that regulate synaptic transmission activities 62. Neuroligins are expressed from four genes (*NLGN1-4*) in mammals, and each one exhibits a distinct synaptic location and function 63. The isoform* NLGN3* is located at both the excitatory and inhibitory synapses. Recent studies have shown that* NLGN3* promotes glioma proliferation through the PI3K-mTOR pathway 64, 65. Moreover, *NLGN3* gene expression was found to be negatively correlated with the overall survival of glioma patients, suggesting the clinical significance of NLGN3 in the diagnosis and prognosis of glioma 64. However, to the best of our knowledge, the expression of NLGN3 in EVs has not been reported. We measured the expression of *NLGN3* in serum EVs from glioma patients (n =18) and healthy individuals (n =9) and found that the mRNA level of* NLGN3* was significantly higher in glioma patients than in healthy donors (**P < 0.01), although the mRNA level of *NLGN3* varied between each glioma patient (Figure 5A). This result was also confirmed by Western blot analyses showing higher expression of NLGN3 protein in some glioma patients compared to the healthy individuals (Figure S10 A, B). These results showed the potential of NLGN3 in detecting glioma. Some studies have suggested that the expression level of NLGN3 was related to the progression and recurrence of glioma 66, 67. However, further investigation is still needed to explore the reason for the large variation in the expression of NLGN3 in individual glioma patients.

We next tested the expression of *PTTG1* in serum EVs from glioma patients. *PTTG* was first identified in rat pituitary tumor cells 68. This gene plays important roles in cell proliferation and transformation, and the protein expressed from *PTTG* (securin) has been shown to be responsible for chromatid separation in mitosis 69. Studies have shown that PTTG is involved in the migration and invasion of glioma, suggesting the role of PTTG as a diagnostic and therapeutic target for glioma 70-73. Increased expression of PTTG in the tumor tissues has been found in various tumors, including glioma 71, 74, and has been shown to be related to the poor prognosis of glioma patients 71. However, studies on the expression of *PTTG1* in EVs from glioma patients have rarely been reported. We found negligible mRNA levels of *PTTG1* in EVs from healthy donors (n =9) and increased *PTT*G1 mRNA in EVs from glioma patients (n =18) (nonparametric test, **P <0.01), although the mRNA level of *PTTG1* varied in glioma patients (Figure 5B). No expression of securin, the protein translated from *PTTG1*, was found in EVs from the glioma patients by Western blot analyses (Figure S10 C, D in supporting information). We attributed this result to the low content of intracellular proteins enclosed in the EVs, which are membrane protein-abundant carriers.

## Discussion

Glioma is the most common malignant brain tumor. The lack of reliable diagnostic strategies for the screening and early diagnosis of glioma is one of the main reasons for the high morbidity and mortality of glioma. EVs are secreted by most cells. Since EVs carry molecular information from source cells, such as nucleic acids and proteins, EVs can be used as biomarkers for the diagnosis of diseases, including cancer 19, 20, 60, 75. The enrichment and detection of EVs in serum enable noninvasive and blood-based diagnosis of cancers and therefore provide an opportunity for the screening and early diagnosis of cancers, including glioma.

In this study, we established an estimation for the enrichment and detection of tumor-derived serum EVs by applying microbead-assisted flow cytometry. This method solved the problem that the nanoscale size of EVs exceeded the detection line of flow cytometry. Using this method, we proved that the expression status of markers in EVs represented well in the parental cells, implying the potential of EVs in diagnosing glioma. We evaluated the diagnostic efficacy of serum EVs for glioma patients and found that EGFR^+^ EVs were able to diagnose glioma patients with high sensitivity and specificity (AUC 0.900, 95% CI 0.8064 ~ 0.9936, *P* <0.0001). We further found that the expression of EGFR in serum EVs could distinguish high-grade glioma patients from low-grade ones, and the level of EGFR in serum EVs correlated well with Ki-67-LI in tumor tissue, a widely accepted marker for the proliferation of glioma, indicating EGFR^+^ EVs as an indicator for the malignancy of glioma. We also investigated the value of mRNA in serum EVs in diagnosing glioma. We investigated the mRNA levels of *NLGN3* and *PTTG1*, two genes related to the proliferation and poor prognosis of glioma, in serum EVs, and found higher mRNA levels of *NLGN3* and *PTTG1* in serum EVs from glioma patients compared to those from normal individuals, although the expression level varies between glioma patients. These results suggest the potential of *NLGN3* and *PTTG1* in EVs in detecting glioma. However, the reason for the variety of the compositions in EVs and the relationship between the variety and tumor progression and recurrence remain to be explored, as it will largely improve the development of biomarkers and diagnostic technologies. Taken together, this study showed the value of serum EVs in the diagnosis and prognosis prediction of glioma, suggesting the great potential of this EV-based assessment in the routine screening, diagnosis and prognosis prediction of glioma. This method also provided opportunities for the monitoring of tumor progression and drug-responses of glioma and would facilitate the management of glioma.

## Supplementary Material

Supplementary figures and tables.

## Abbreviations

AUC: area under the curve
CNS: central nervous system
CSF: cerebrospinal fluid
CTCs: circulating tumor cells
ctDNA: circulating tumor DNA
CXCL12: chemokine stromal cell derived factor-1
CXCR4: C-X-C chemokine receptor 4
EGFR: epidermal growth factor receptor
EVs: extracellular vesicles
FGF 2: fibroblast growth factor 2
HSA: human serum albumin
IHC: immunohistochemical
LI: labeling index
MGMT: O6-methylguanine DNA methyl transferase
NLGN3: neuroligin 3
NSCLC: non-small-cell lung cancer
PTEN: gene of phosphate and tension homology deleted on chromsome ten
PTTG 1: pituitary tumor transforming gene 1
