# Supplementary material for: Evaluation of serum extracellular vesicles as noninvasive diagnostic markers of glioma
Source: Theranostics. 2019 Jul 9;9(18):5347–58. doi: 10.7150/thno.33114 (PMC6691576; doi:10.7150/thno.33114)
Supplement: Supplementary file 1 — Supplementary figures and tables. [file thnov09p5347s1.pdf]

Supporting Information

Evaluation of serum extracellular vesicles as the noninvasive diagnostic markers of glioma

Huayi Wang[1,3,5], Dengzhi Jiang[2], Wenzhe Li[1,6], Xiang Xiang[2], Jun Zhao[2], Bin Yu[4], Chen Wang[1,5], Zhaohui He[2,*], Ling Zhu[1,5,*], Yanlian Yang[1,5,*]

[1] CAS Key Laboratory of Standardization and Measurement for Nanotechnology, CAS Key Laboratory of Biological Effects of Nanomaterials and Nanosafety, CAS Center for Excellence in Nanoscience, National Center for Nanoscience and Technology, Beijing 100190, China

[2] Department of Neurosurgery, First Affiliated Hospital of Chongqing Medical University, 1 Friendship Road, Chongqing 400016, China

[3] Department of Chemistry, Tsinghua University, Beijing, 100084, China

[4] Department of Radiology, First Affiliated Hospital of Chongqing Medical University, 1 Friendship Road, Chongqing 400016, China

[5] University of Chinese Academy of Sciences, 19 A Yuquan Rd, Shijingshan District, Beijing 100049, China

[6] Academy for Advanced Interdisciplinary Studies, Peking University, Beijing 100871, China

Corresponding authors: Zhaohui He (geno_he@163.com), Ling Zhu (zhul@nanoctr.cn), Yanlian Yang (yangyl@nanoctr.cn)

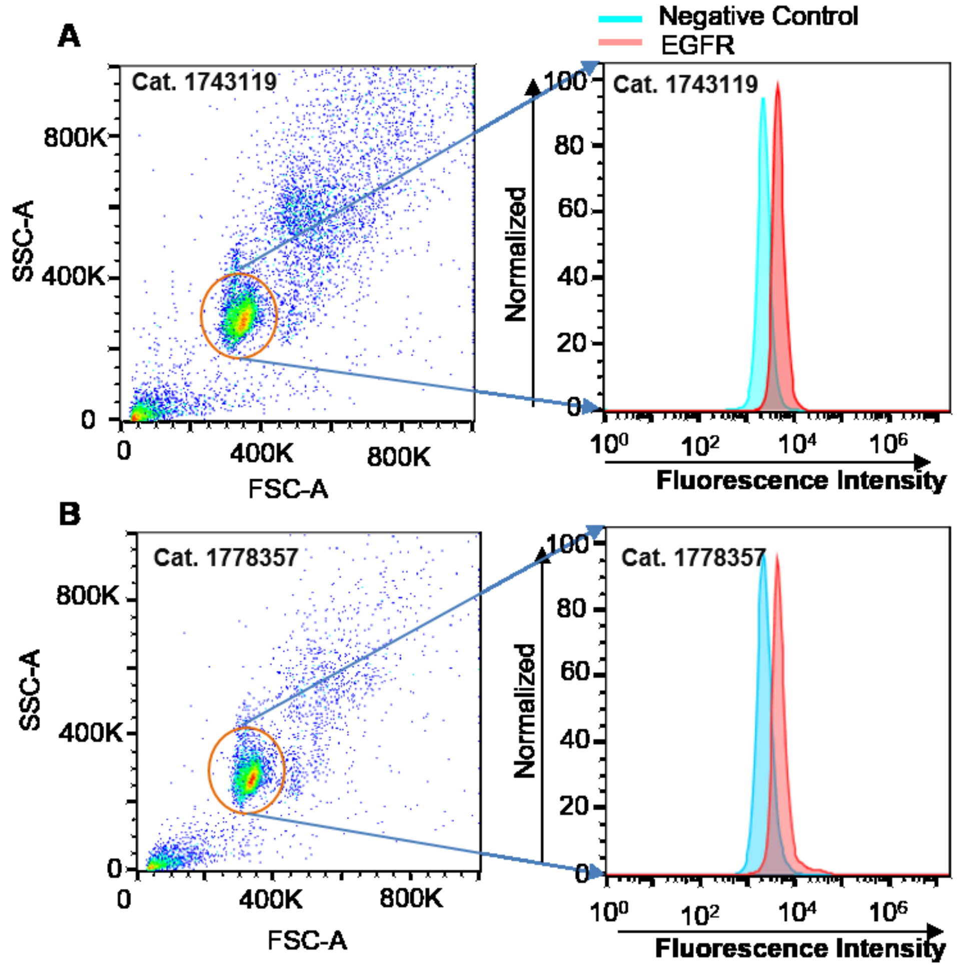

Figure S1. Evaluation of the consistency of the aldehyde latex beads. EVs extracted from the same glioma patient were mixed with different batches of aldehyde latex beads (Cat. 1743119 (A) and Cat. 1778357 (B)) at the same ratio (4 μg EVs /1μL beads). The captured EVs were labeled with anti-EGFR and the fluorescent secondary antibody. Flow cytometry analysis showed that the two batches of beads exhibited similar results on the expression of EGFR in the EVs.

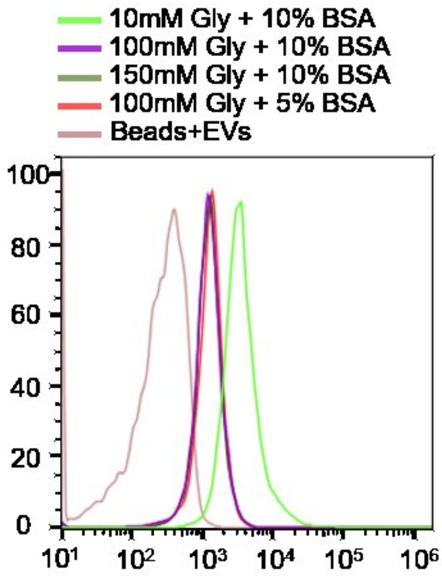

Figure S2. Optimization of the formula of the blocking buffer for flow cytometry analysis. The blocking effect of different formulas of the blocking buffer (10 mM Glycine with 10% BSA, 100 mM Glycine with 10% BSA, 150 mM Glycine with 10% BSA, 100 mM Glycine with 5% BSA) was compared. 10% BSA in 100 mM or 150 mM glycine exhibited almost the same blocking effect that was much better than the one of 10 mM glycine, indicating that the glycine concentration of 100 mM is a relatively desired concentration with good blocking effect and without overblocking. 100 mM Glycine with 10% or 5% BSA exhibited almost the same blocking effect, indicating that 10% BSA would not cause overblocking. This experiment was carried out using EVs from one glioma patient (4μg EVs:1 μL beads) and secondary antibody (anti-rat, ab150157, abcam, dilution 1/1000).

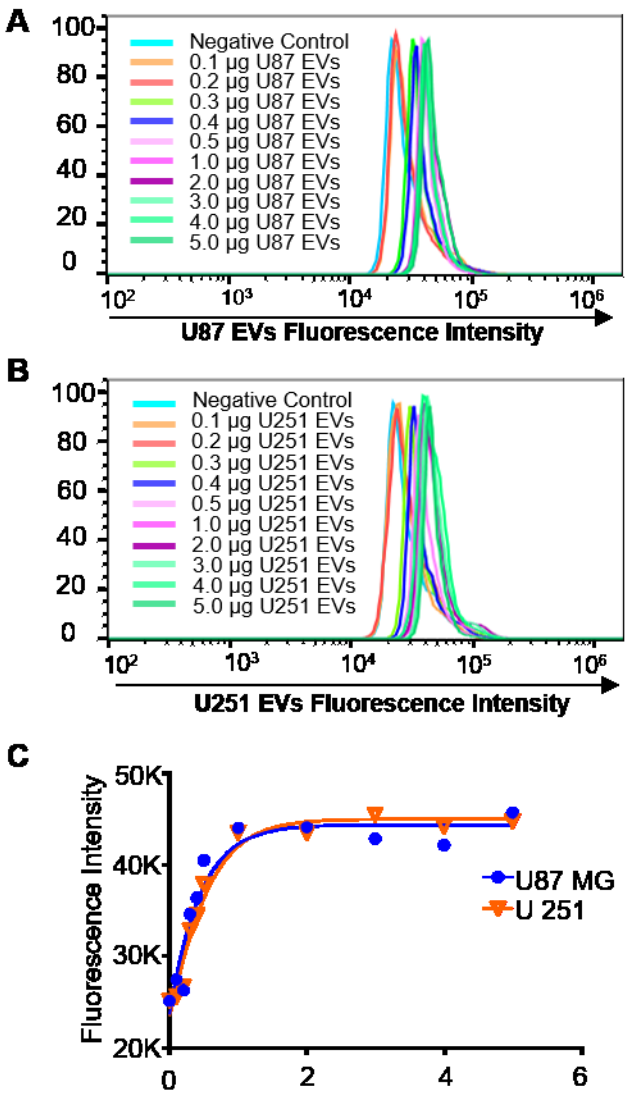

Figure S3. Saturation assay of the enrichment of EVs on the aldehyde latex beads. (A) Flow cytometry analysis of the binding of different amount of EVs from U87MG cells on 1 µL beads. (B) Flow cytometry analysis of the binding of different amount of EVs from U251 cells on 1 µL beads. (C) Saturation curve of the binding of EVs on the beads. Saturation concentration is about 1 µg EVs/ µL beads. The enrichment efficiency is

determined to be ～$7.6*10^{-13}$ g EVs/bead calculated by dividing the saturation concentration of EVs on the beads with the number of beads in the solution.

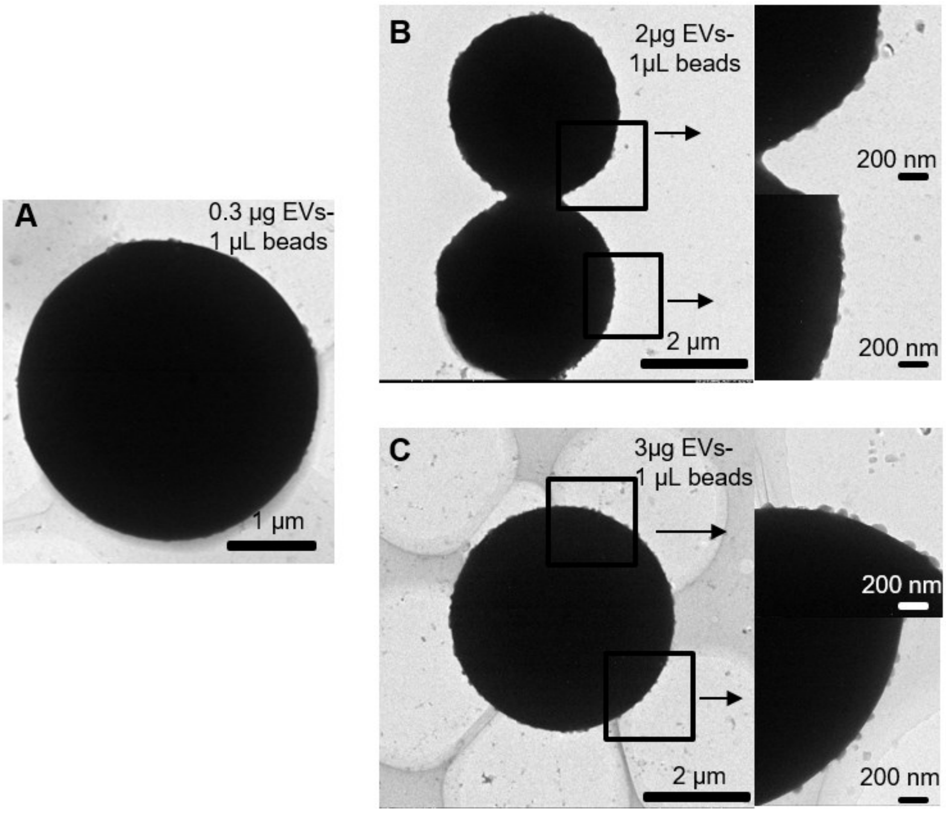

Figure S4. TEM characterization of the EVs bound on the aldehyde latex beads at different EV/bead ratio. (A) 0.3 µg EVs /1µL beads, (B) 2 µg EVs /1µL beads, (C) 3 µg EVs /1µL beads.

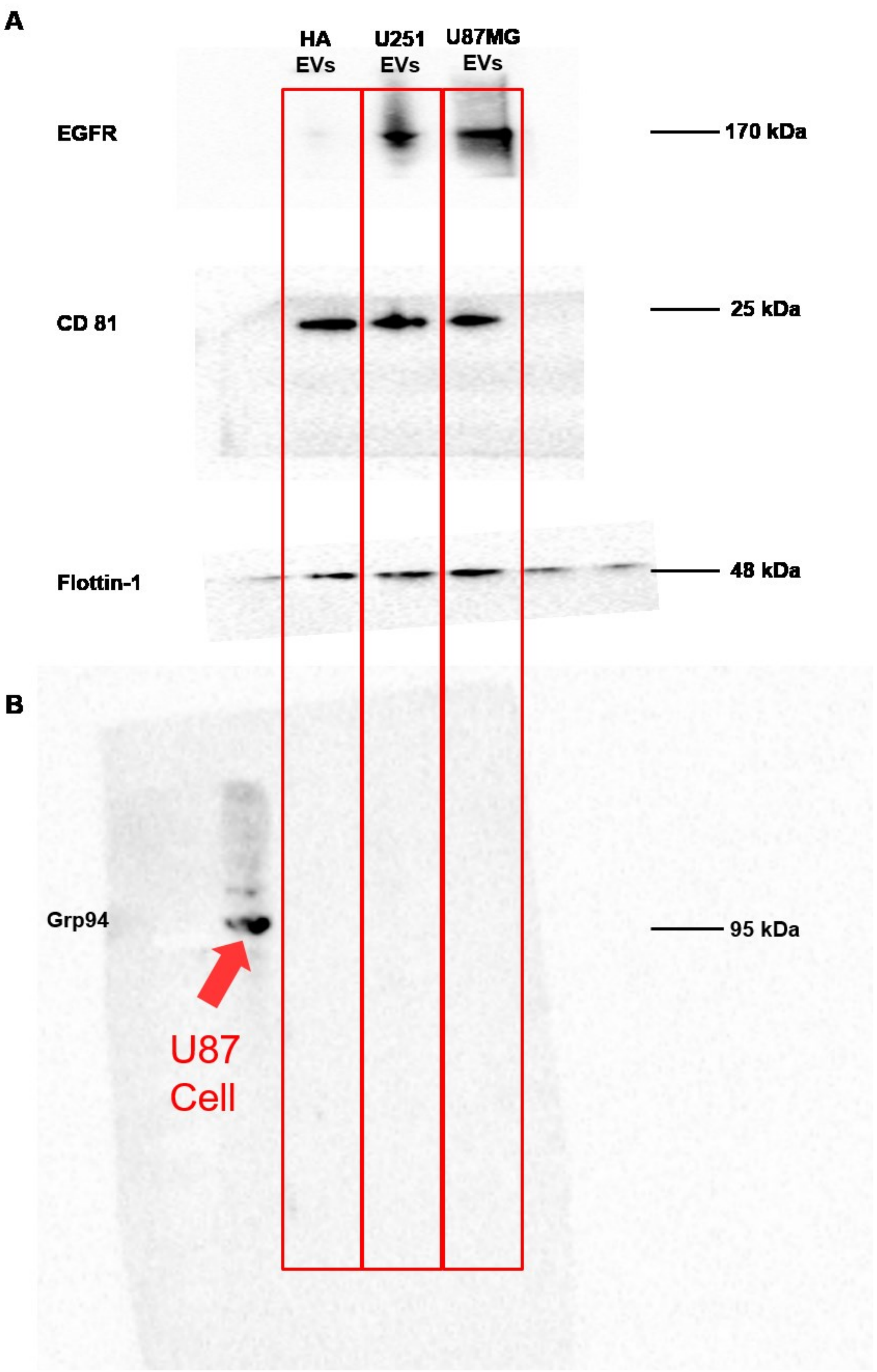
A
HA EVs
U251 EVs
U87MG EVs
EGFR
170 kDa
CD 81
25 kDa
Flottin-1
48 kDa
B
Grp94
95 kDa
U87 Cell

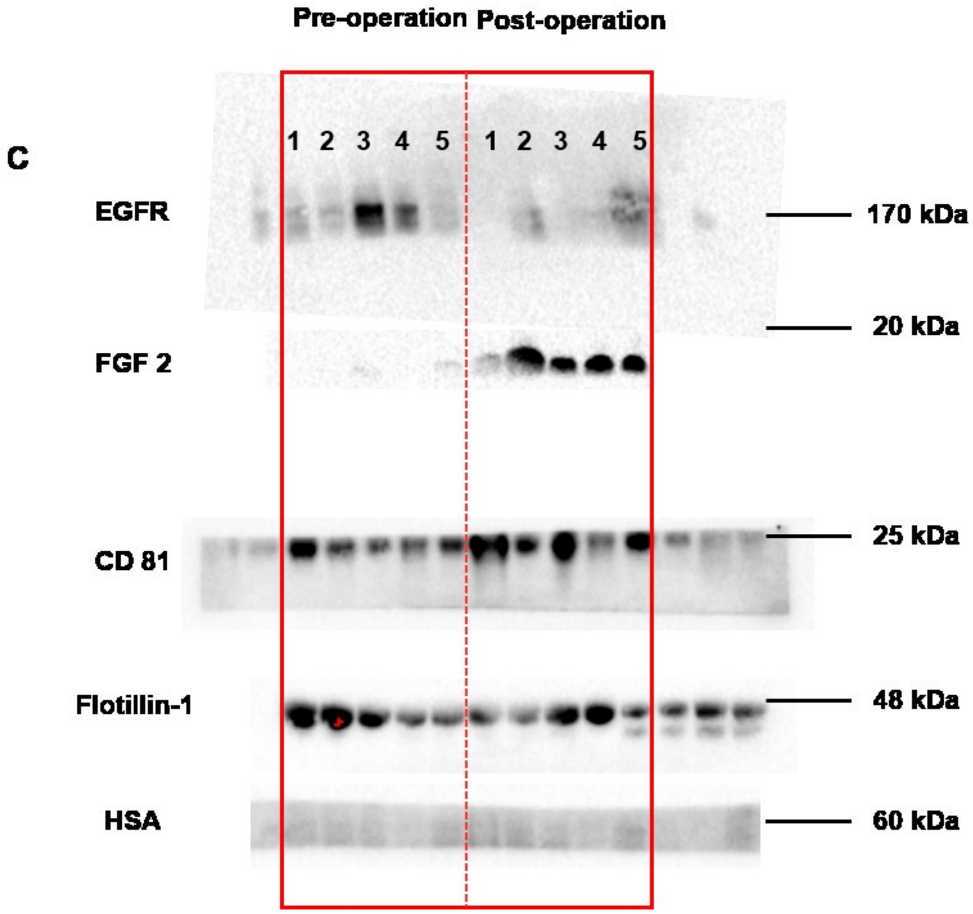
Pre-operation
Post-operation
C
1 2 3 4 5
1 2 3 4 5
EGFR
FGF 2
CD 81
Flotillin-1
HSA
170 kDa
20 kDa
25 kDa
48 kDa
60 kDa

D

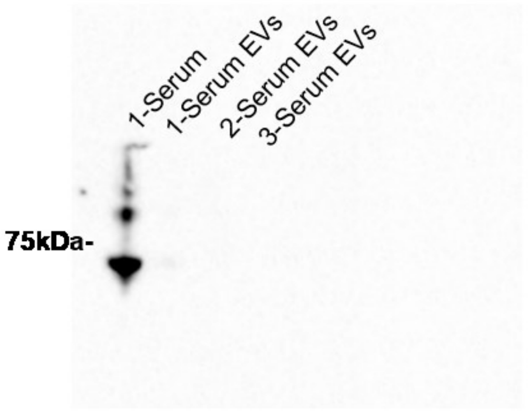

E

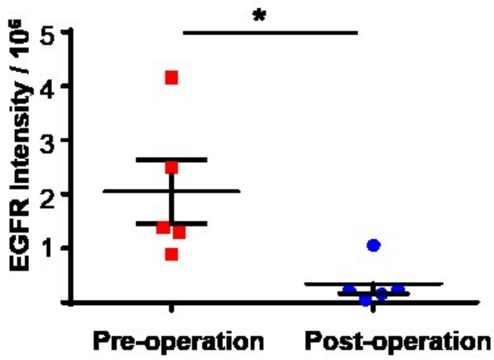

Figure S5.

(A) Uncropped full-length blots of the expression of EGFR, CD81and Flotillin-1 in EVs of human astrocytes and human glioma cells (U251 and U87MG). (B) Western blotting of the expression of Grp94 in the cell lysates from U87MG, and in the EVs extracted from HA, U251 and U87MG. (C) Expression of EGFR, FGF 2, CD 81, Flotillin-1, HSA in EVs from human serum. (D) Western blotting of the expression of HSA in human serum and EVs extracted from human sera. (E) Quantification of the EGFR expression levels on serum EVs comparing before and after surgery using EGFR Western blots.

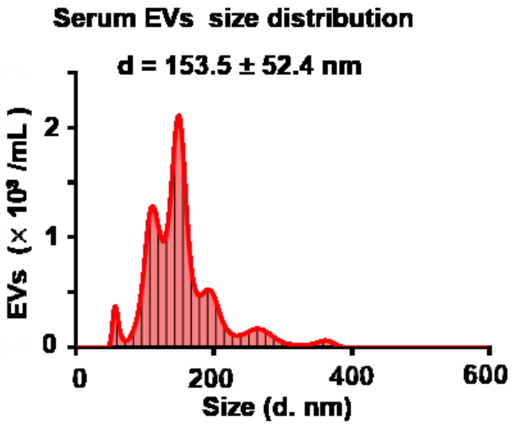

Figure S6. Size distribution of the EVs extracted from the sera of glioma patients as analyzed by nanoparticle tracking analysis (NTA).

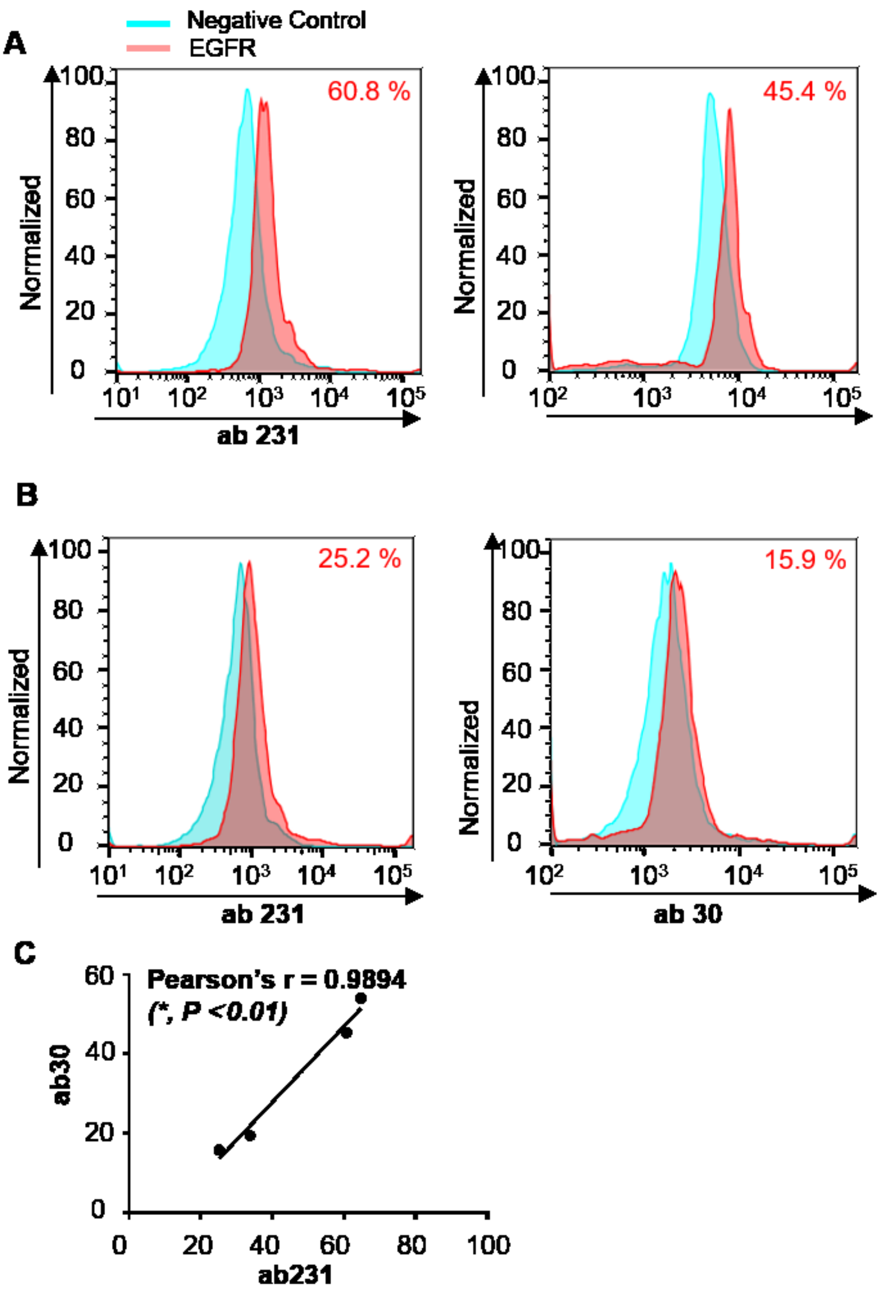

Figure S7. Evaluation of the influence of antibodies on flow cytometry analysis of EV. (A) and (B) Flow cytometry analysis of the same sample using different antibodies ab231 and ab30 (Abcam). (C) Correlation analysis of the measurements from the two antibodies. (Pearson correlation analysis $r = 0.9894$, * $P < 0.01$)). (Detailed data in Table S4)

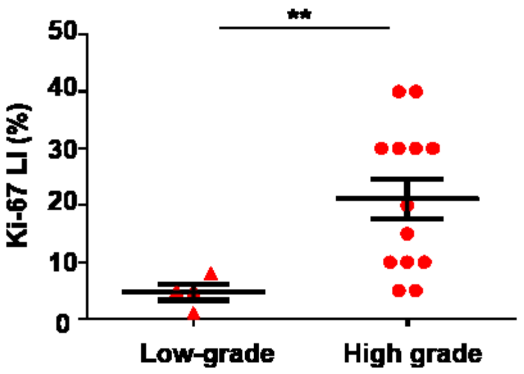

Figure S8. Ki-67 LI expression difference between Low-grade glioma and high-grade glioma. (non-parametric test， **P = 0.0071)

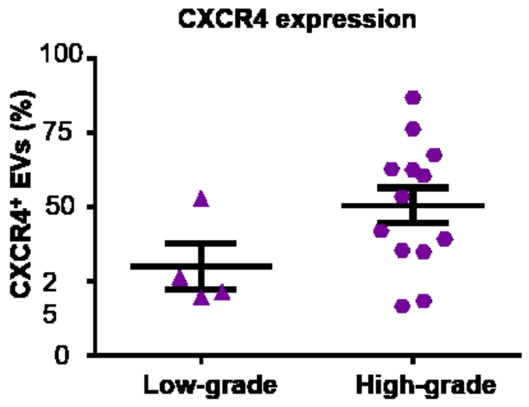

Figure S9. Percentage of $CXCR4^+$ EVs in low-grade (n = 4) and high-grade (n = 13) glioma patients measured by flow cytometry.

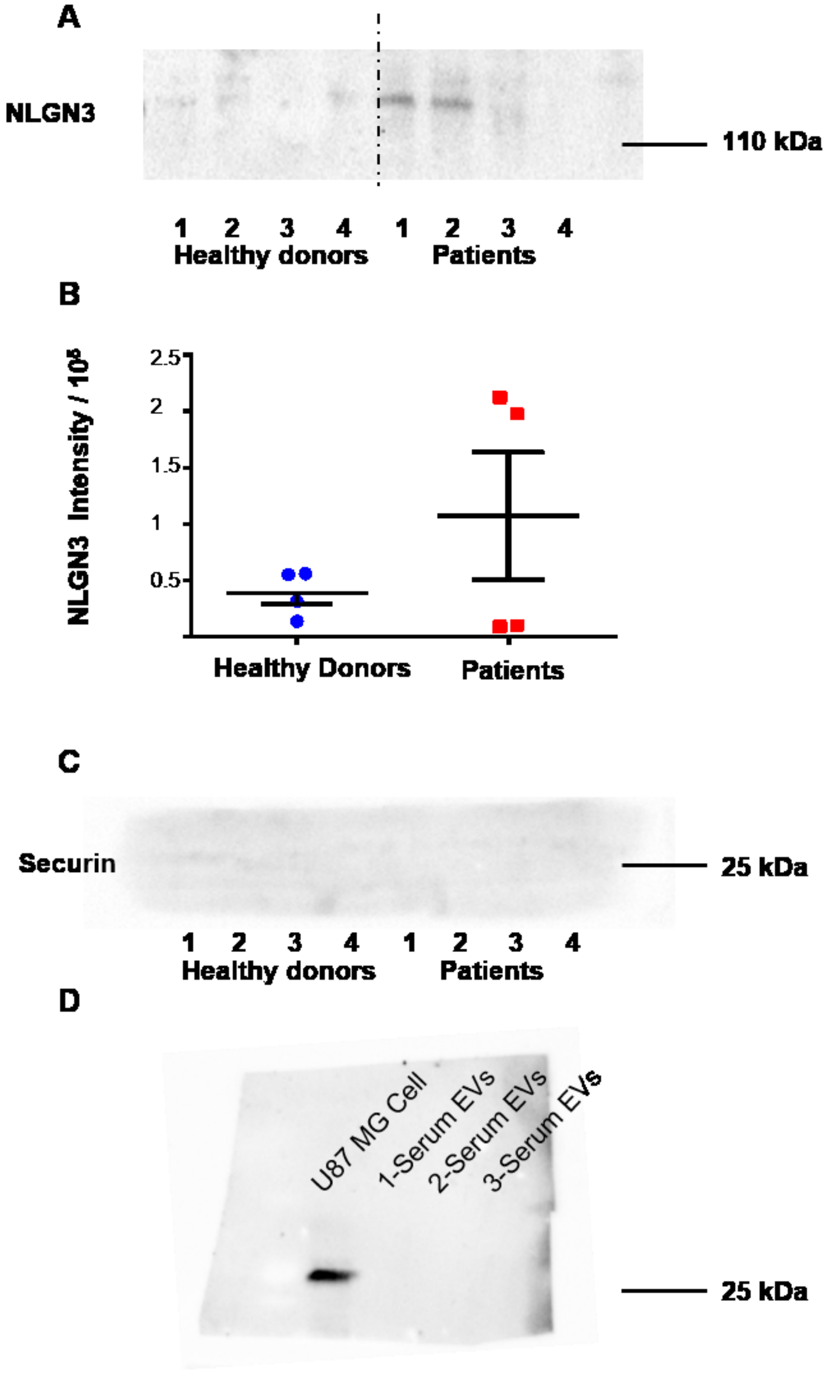

Figure S10. (A)Western blots expression of NLGN3. (B) Quantification of the NLGN3 expression levels on serum EVs comparing healthy donors and patients on NLGN3

Western blots. (C) Western blots expression of Securin. (D) Western blots of the expression of Securin in cells and EVs extracted from human sera.

Table S1. Clinical information for patient profiling study. Tumor size are measured based on MRI and CT. Patients who did not undergo tumor resection have no pathology diagnosis.

Sample#	Age	Gender	WHO	KI-67 LI	Tumor Size	Pathology Diagnosis
1	42	F	II	1	5.2cm×6.3cm×3.5cm	Neuroastrocytoma
2	43	F	II	5	4.0cm×4.0cm×3.0cm	Oligodendroglioma
3	48	M	II	8	6.5cm×4.3cm×5.5cm	Oligodendroglioma
4	37	F	II	5	Diffuse glioma	Oligodendroglioma
5	53	M	III	5	4.9cm×4.0cm×4.0cm	Anaplastic gliomas
6	48	M	III	5	4.0cm×3.0cm×4.0cm	Anaplastic astrocytoma
7	54	M	III	10	2.8cm×2.8cm×4.1cm	Anaplastic astrocytoma
8	48	M	III	10	6.8cm×5.4cm×5.1cm	Oligodendroglioma
9	60	F	III	10	3.7cm×2.6cm×4.0cm	Neuroastrocytoma
10	68	M	III	30	4.1cm×4.5cm×7.1cm	Anaplastic astrocytoma
11	53	F	III	40	5.1cm×4.1cm×4.1cm	Anaplastic gliomas
12	52	M	III	40	6.3cm×3.2cm×5.0cm	Oligodendroglioma
13	52	F	IV	30	7.9cm×4.2cm×5.4cm	Glioblastoma
14	32	M	IV	30	3.6cm×2.1cm×4.0cm	Glioblastoma
15	60	F	IV	15	5.1cm×4.3cm×4.5cm	Glioblastoma
16	79	M	IV	30	4.2cm×3.7cm×4.0cm	Glioblastoma
17	65	F	IV	20	6.0cm×4.5cm×4.0cm	Glioblastoma
18	62	M	N	N	3.2cm×2.5cm×2.5cm	N
19	45	M	N	N	8.1cm×6.2cm×5.1cm	N
20	53	F	N	N	4.4cm×3.9cm×4.0cm	N
21	47	M	N	N	8.6cm×7.7cm×6.5cm	N
22	37	M	N	N	4.6cm×3.5cm×3.5cm	N
23	59	M	N	N	1.9cm×2.2cm×1.7cm	N

Table S2. Information of patients enrolled in the study.

Variable	Glioma	Controls
Number of samples	23	12
Age	32-79	45-83
Mean	51.95	59.33
Male	14	9
Female	9	3
WHO grade		
WHO II	4	
WHO III	8	
WHO IV	5	

Table S3. Fluorescent intensities of bead-bound EVs at different EV/bead ratio as measured by flow cytometry.

Evs Protein/beads (µg/µL)	Median Fluorescence Intensity of U87	Median Fluorescence Intensity of U251
0.0	25154.	25154.
0.1	27461.	25696.
0.2	26313.	26690.
0.3	34649.	32887.
0.4	36419.	34567.
0.5	40522.	37918.
1.0	44030.	43614.
2.0	44135	43511
3.0	42896.	45517.
4.0	42189.	44239.
5.0	45733.	44874.

Table S4. Flow cytometry analysis of the consistency between different two antibodies.

	ab231	ab30
1	60.8	45.4
2	64.8	54.1
3	33.8	19.5
4	25.2	15.9

Table S5. Percentage of EGFR$^+$ EVs in paired glioma patients pre- and post-operation measured by flow cytometry.

	Pre-Operation	Post-Operation	Difference value	Decreasing ratio
1	72.7	48.4	24.3	0.33
2	76	62.8	13.2	0.174
3	31.6	17.9	13.7	0.434
4	91.6	20.3	71.3	0.778
5	41.8	23.9	17.9	0.428
6	42.7	12	30.7	0.719
7	25.6	12.6	13	0.508
8	10.1	8.6	1.5	0.149
Average				0.440
SD				0.213

Table S6. Receiver operating characteristic (ROC) analysis of the discriminative efficacy of $EGFR^+$ EVs and total protein concentration in EVs in distinguishing glioma patients and normal individuals.

	Area	Std. Error	95% confidence interval	P value
$EGFR^+$ EVs expression	0.9000	0.04774	0.8064 to 0.9936	< 0.0001
EVs protein concentration	0.5109	0.1030	0.3089 to 0.7128	0.9170

Table S7. Pearson correlation analysis of the $EGFR^+$ EVs and KI-67 LI in tissue of glioma patients.

Pearson r	
r	0.8078
95% confidence interval	0.5347 to 0.9281
R square	0.6525
P value	
P (two-tailed)	< 0.0001
P value summary	****
